# Topical application of bFGF on acid-conditioned and non-conditioned dentin: effect on cell proliferation and gene expression in cells relevant for periodontal regeneration

**DOI:** 10.1590/1678-7757-2017-0051

**Published:** 2017

**Authors:** Fernanda Regina Godoy Rocha, João Antônio Chaves de Souza, Morgana Rodrigues Guimarães-Stabili, José Eduardo Cezar Sampaio, Carlos Rossa

**Affiliations:** 1 University of Florida at Gainesville, Department of Oral Biology and Periodontology, Gainesville, USA; 2 Universidade Federal de Goiás, Faculdade de Odontologia, Departmento de Periodontia, Goiania, GO, Brasil; 3 Universidade de São Paulo, Faculdade de Odontologia, Departmento de Estomatologia, São Paulo, SP, Brasil; 4 Univ Estadual Paulista - UNESP, Faculdade de Odontologia de Araraquara, Departmento de Diagnóstico e Cirurgia, Araraquara, SP, Brasil

**Keywords:** Intercellular signaling peptides and proteins, Periodontal ligament, Dental cementum, Bone marrow, Cells

## Abstract

Periodontal regeneration is still a challenge in terms of predictability and magnitude of effect. In this study we assess the biological effects of combining chemical root conditioning and biological mediators on three relevant cell types for periodontal regeneration.

**Material and Methods:**

Bovine dentin slices were conditioned with 25% citric acid followed by topical application of basic fibroblast growth factor (bFGF, 10 and 50 ng). We used ELISA to assess the dynamics of bFGF release from the dentin surface and RT-qPCR to study the expression of Runx2, Col1a1, Bglap and fibronectin by periodontal ligament (PDL) fibroblasts, cementoblasts and bone marrow stromal cells (BMSC) grown onto these dentin slices. We also assessed the effects of topical application of bFGF on cell proliferation by quantification of genomic DNA.

**Results:**

Acid conditioning significantly increased the release of bFGF from dentin slices. Overall, bFGF application significantly (p<0.05) increased cell proliferation, except for BMSC grown on non-conditioned dentin slices. Dentin substrate discretely increased expression of Col1a1 in all cell types. Expression of Runx2, Col1a1 and Fn was either unaffected or inhibited by bFGF application in all cell types. We could not detect expression of the target genes on BMSC grown onto conditioned dentin.

**Conclusion:**

Acid conditioning of dentin improves the release of topically-applied bFGF. Topical application of bFGF had a stimulatory effect on proliferation of PDL fibroblasts, cementoblasts and BMSC, but did not affect expression of Runx2, Col1a1, Bglap and fibronectin by these cells.

## Introduction

Evidence indicates that current cause-related periodontal therapy is effective in stopping disease progression and reducing local inflammation, but regeneration of periodontal tissues lost as a consequence of disease still poses a challenge both in terms of predictability and magnitude of effect^5^. Several studies^2^ ^,^ ^17^ ^,^ ^24^ have evaluated the potential of a host of biological and chemical mediators, particularly growth and differentiation factors, to improve the results of regenerative therapy. Acid root conditioning is one of the first therapeutic approaches developed with this intent^4^ ^,^ ^7^. There is great controversy in regard to the conditioning agents and methods of application.

In fact, there are products currently available commercially for applications in periodontal regeneration based on the biological properties of growth factors. One such product includes in its surgical procedure protocol acid conditioning of the root surface to remove smear layer from the dentin^4^ and there is evidence indicating that chemical conditioning with EDTA improved PDGF-induced adhesion of periodontal ligament fibroblasts in comparison with the use of PDGF without previous EDTA conditioning^3^.

Chemical conditioning of dentin has also been extensively studied and may favor the repair/regeneration of periodontal tissues. Modifications on dentin surface induced by chemical conditioning, including removal of smear layer, greater exposure of collagen fibers and increased adhesion of blood cells and fibrin clot^8^ ^,^ ^16^ may favor the adsorption of topically applied growth factors and also turn the dentin surface more conductive to cell attachment to promote blood clot attachment.

Our research group has systematically evaluated the most commonly used conditioning agents (citric acid, tetracycline hydrochloride and EDTA), optimizing conditions for their topical use based on the effectiveness of smear layer removal, exposure of dentin collagen fibrils and adhesion of blood clot and cells^6^ ^,^ ^11^ ^,^ ^16^ ^,^ ^23^. These *in vitro* studies indicated that citric acid is the best candidate for a chemical conditioning agent to improve removal of smear layer, exposure of collagen fibers and adhesion of blood cells and fibrin clot. Other possible advantages of chemical root conditioning include: 1) the exposure of endogenous growth factors and biological mediators stored in the dentin or cementum matrix; and 2) the increased adsorption of exogenous growth factors topically applied to the root surfaces due to the exposed collagen fibrils of the dentin matrix.

There are reports of promising results for periodontal regeneration with the use of root conditioning in pre-clinical *in vivo* models, but the results of clinical studies using root conditioning as a sole technique or even combined with other techniques are, in general, disappointing^1^ ^,^ ^18^ ^,^ ^28^. However, very few studies have assessed the combination of chemical root/dentin conditioning with topical application of biologically-active mediators. Polypeptide growth factors may act locally or systemically and modulate several cell functions, including proliferation, migration, chemotaxis, differentiation, and gene expression^12^. Fibroblast growth factor 2 or basic fibroblast growth factor (bFGF) is a growth factor with biological effects that may have important implications for periodontal regeneration, namely induction of angiogenesis, cell proliferation and differentiation, resulting in increased extracellular matrix production^15^. bFGF may be useful for periodontal regeneration, because of its angiogenic properties and also the chemotactic and proliferative effects on PDL cells, which may improve healing and regeneration processes. Both pre-clinical studies^19^ ^,^ ^21^ ^,^ ^25^ and one clinical study^14^ assessed the use of bFGF in various concentrations and modes of application, suggesting that bFGF may enhance periodontal regeneration. This study addresses the hypothesis that root conditioning improves the biological effect of bFGF topically applied onto the dentin surface.

## Material and methods

### Preparation of dentin slices

Rectangular dentin slices of 1.0×0.5×0.3 cm (length × width × heigth) were obtained manually from the roots of bovine incisors, by sectioning 2-3 mm apically to the cementum enamel junction with a diamond disc mounted on a slow speed dental handpiece. The portion of the dentin facing the pulp was made fiat by using aluminum oxide disks (medium, fine and extra-fine) mounted on a handpiece and used at low speed. The external surface (i.e., facing the periodontal ligament and bone) of the dentin slices was submitted to 5 scaling strokes by a single operator using a 5-6 Gracey curette. Cells were always seeded onto this external surface (i.e., submitted to the manual scaling) of the dentin slices with an area of 50 mm^2^. After preparation, the dentin slices were then sterilized by ethylene oxide gas to avoid changes to the microstructure of the dentin or accumulation of chemical residues on the dentin surface that could occur by other means of sterilization.

### Cell lines

The murine periodontal ligament fibroblasts cell line (PDL) and cementoblast cell line (OCCM) were kindly provided by Dr. Martha J. Somerman, National Institutes of Health/National Institute of Dental and Craniofacial Research (NIH/NIDCR), Bethesda, MD^9^. The primary murine bone marrow stromal cells (BMSCs) were obtained from bone marrow flushings of long bones (tibia and femur) of C57BL/6 mice. These cells were maintained in Dulbecco's Modified Eagle Medium (DMEM, Life Technologies, Thermo Fisher Scientific, Waltham, MA, USA), supplemented with 10% volume to volume (v:v) heat-inactivated fetal bovine serum (FBS, Life Technologies, Thermo Fisher Scientific, Waltham, MA, USA), and penicillin (100 IU/mL)/streptomycin (100 μg/mL) (Life Technologies, Thermo Fisher Scientific, Waltham, MA, USA) at 37°C in an atmosphere containing 5% CO_2_. For the experiments, cells were plated in a sufficient density (determined in preliminary experiments-data not shown) to achieve 80% confluency over 48 h, enzymatically detached (Tryple, Life Technologies, Thermo Fisher Scientific, Waltham, MA, USA) and resuspended in low-FBS (2% v:v) culture medium (DMEM) at 1×10^6^ cells/mL. Fifty μL of this cell suspension (5×10^4^ cells) were seeded onto the dentin slices or on the control wells and allowed to attach for 1 h before adding the Anal volume of culture medium (which varied according to the specific experiment/well size). This seeding procedure aimed at obtaining similar cell densities in dentin slices and cell culture plastic, as cell density and cell-to-cell contact may influence the response to the stimulus with the growth factor.

### Experimental procedures

The experimental conditions were the following: 1) Control (cells on dentin slices without any growth factor); 2) 10 ng of bFGF (R&D Systems, Minneapolis, MN, USA) applied topically; 3) 50 ng of bFGF applied topically. For each of these conditions we had 12 dentin slices. Of these 12 dentin slices for each experimental condition, 6 were submitted to 25% citric acid conditioning for 3 minutes, followed by a wash with 10 mL of sterile phosphate buffered saline (PBS, pH 7.2, Life Technologies, Thermo Fisher Scientific, Waltham, MA, USA) before the seeding of cells (experimental condition #1) or before topical application of bFGF and seeding of cells (experimental conditions #2 and #3). Having 12 dentin slices (6 without citric acid conditioning, 6 with citric acid conditioning) for each experimental condition allows for two replicates with each cell type (PDL, OCCM or BMSC). All experiments were repeated independently (using different cell passages of PDL and OCCM cells, and BMSC obtained from different animals) three times.

Dentin slices were placed into 12-well cell culture plates (1 slice/well). Topical application of the growth factors was carried out by preparing a suspension of 50 μL of DMEM (without FBS and antibiotics) containing the desired concentration of the growth factor(s). The 50 μL volume yielded optimal conditions to completely cover the dentin slices. This suspension of DMEM containing the growth factors was removed by aspiration after 2 minutes. Immediately, 50 μL of a 1×10^6^ cells/mL suspension of each cell line was pipetted onto the dentin slices and placed in a CO_2_ incubator at 37°C for 4 hours for initial cell adhesion. After the initial 4-hour period, 1.5 mL of DMEM supplemented with 2% FBS were added to each well, and they were incubated for a further 20-hour period. In the negative control wells (without dentin slices), the same quantity of cells was plated and the growth factors added directly to the culture medium. Cells were collected mechanically with sterile plastic cell scrapers. The exact same procedure was used to plate the cells for the tissue culture-treated plastic substrate controls in which no dentin slice was used as substrate.

### Release of bFGF from the dentin substrate analysis

We prepared dentin slices using the exact same procedure described under “experimental procedures”, except for the seeding of the cells. After topical application of bFGF (both with and without citric acid conditioning), the dentin slices were placed in 12-well tissue culture plates and 2 mL of non-supplemented DMEM was added to each well. Controls included the addition of bFGF directly to the non-supplemented DMEM to a Anal concentration of 50 ng/mL. Aliquots of 200 uL of the culture medium were collected from wells after 10, 30, 60, 120, 240 e 480 minutes of incubation, and the concentration of bFGF was assessed by ELISA (R&D Systems, Minneapolis, MN, USA). Three independent experiments were performed, and all samples were analyzed in duplicate.

### Cell proliferation analysis

Cells were plated onto 12-well plates directly on cell culture-treated plastic or on dentin slices according to each experimental group, as described before. Genomic DNA was quantitated from the cells harvested by mechanically scraping the dentin slices/bottom of tissue culture wells in all experimental groups. These samples were collected at 24, 48 and 72 h after plating. Genomic DNA was isolated using the Easy-DNA (Life Technologies, Thermo Fisher Scientific, Waltham, MA, USA), according to the manufacturer's instructions. The concentration of DNA in all samples was measured using a nanoscale spectrophotometer (Nanovue Plus, GE Healthcare, Marlborough, MA, USA) and all experiments were performed independently three times.

### Gene expression analysis

Total RNA was isolated using affinity columns and treated with DNase (RNAqueous kit-4-PCR, Ambion Inc., Thermo Fisher Scientific, Waltham, MA, USA). Concentration and purity of total RNA were determined by A260 and A260/A280, respectively, in a UV spectrophotometer for microvolumes. cDNA was synthesized from 100 ng of total RNA using random hexamers primers and moloney leukemia virus reverse transcriptase, according to the instructions of the supplier (High Capacity cDNA synthesis kit, Applied Biosystems, Thermo Fisher Scientific, Waltham, MA, USA). The qPCR reaction was performed using TaqMan chemistry (TaqMan Fast Universal PCR Master Mix, Applied Biosystems, Thermo Fisher Scientific, Waltham, MA, USA) and pre-designed and optimized sets of primers and probes (TaqMan Gene Expression Assays, Applied Biosystems, Thermo Fisher Scientific, Waltham, MA, USA) for detection of target genes (Col1a1, FN1 and Runx2) and the housekeeping gene (GAPDH) on a StepOne Plus thermocycler (Applied Biosystems, Thermo Fisher Scientific, Waltham, MA, USA). All samples were analyzed in duplicate and the cycle threshold (Ct) value of each well was determined. Data was normalized and analyzed by Δ(ΔCt) method using the thermocycler's software. Results are expressed as fold change compared to negative control (cells grown on tissue culture-treated plastic in 12-well plates without stimulation with growth factors).

### Statistical analysis

The statistical analysis was done using GraphPad 5.0 (GraphPad Software Inc., San Diego, CA, USA). Central tendency and dispersion measures were calculated from different experiments. Pairwise comparisons between experimental conditions were performed using non-paired Student's t-test, considering each experimental condition as an independent event. Significance level was 95% (p<0.05) for all analysis.

## Results

### Effect of acid conditioning on the release of bFGF from the dentin substrate

**Figure 1:**
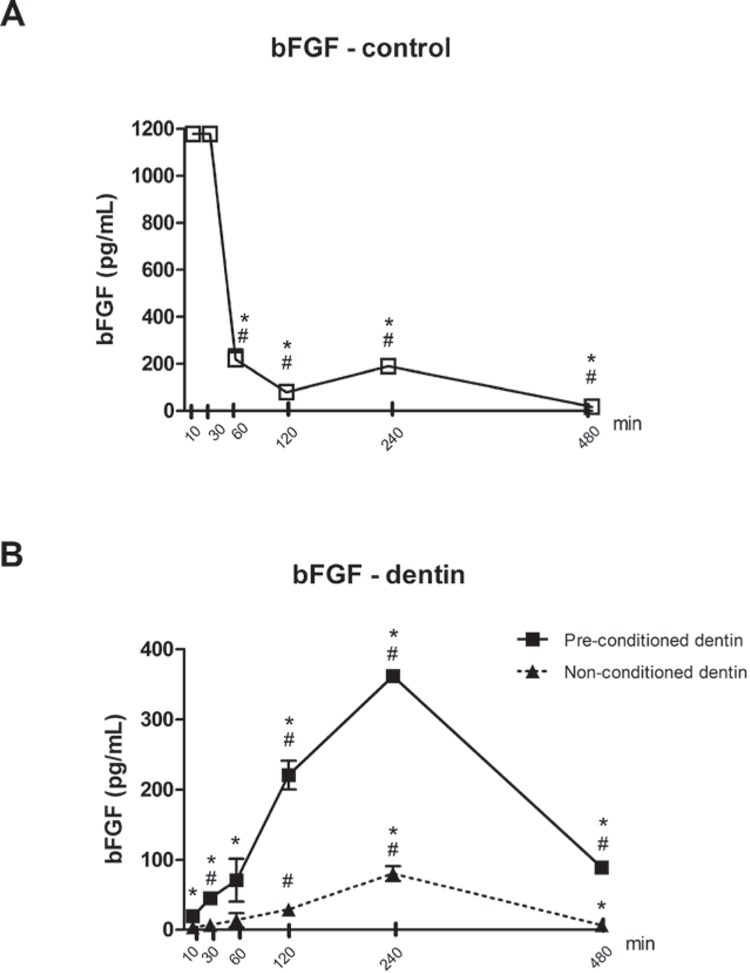
Concentration of bFGF in acqueous-based cell culture medium, after 10 to 480 min of incubation. 50 ug/mL of bFGF was applied topically to dentin slices with and without previous acid conditioning and the quantity of bFGF in the medium was determined in aliquots of the culture medium by ELISA at the indicated periods. In positive control experiments (without dentin slices), the same concentration of bFGF was added directly to the culture medium. Bars and vertical lines indicate means and standard deviations of three independent experiments, assayed in duplicate (* indicates p<0.05 *vs* previous period; # indicates p<0.05 *vs* initial period of assessment)

Acid conditioning previously to the topical application of the growth factor increased the concentration of bFGF released into the culture medium at 10 and 30 minutes. Concentration of bFGF released into the culture medium from dentin samples was lower than that measured in the positive control samples, in which bFGF was added directly to the culture medium, to account for the possible influences of the culture medium, tissue culture plastic or of the spontaneous degradation of bFGF in its detection by ELISA. The very distinct profile of the concentrations detected over the experimental periods indicates a quick degradation of the bFGF in culture medium (at 1 h, the concentration in the culture medium dropped by 6-fold - Figure 1A). The results suggest that some of the growth factor applied onto the dentin substrate was either strongly bound to the substrate and not released from it over 30 minutes; or that it was inactivated (Figure 1).

### Effect of bFGF and culture substrate on proliferation of PDL fibroblasts, cementoblasts and BMSC

**Figure 2:**
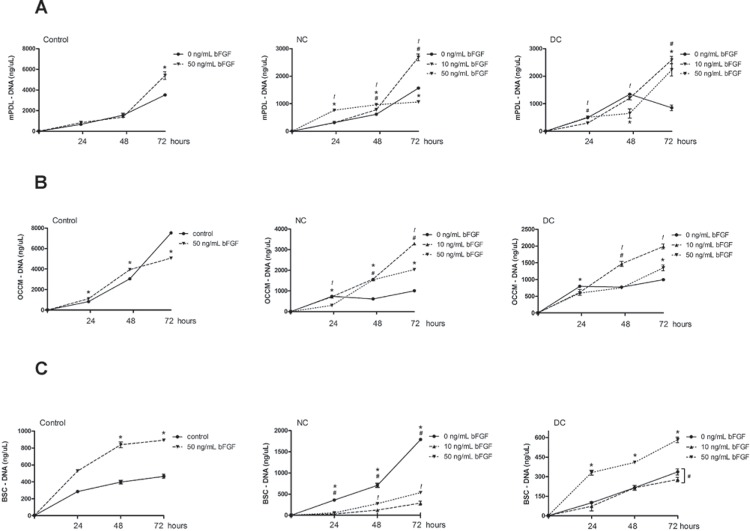
Genomic DNA quantitation indicating the proliferation of murine periodontal ligament fibroblasts (mPDL, panel “A”), cementoblasts (OCCM, panel “B”) and bone marrow stromal cells (BMSC, panel “C”), according to the experimental conditions. Cells were plated onto dentin slices on which bFGF (10 and 50 ng) was applied topically both with and without previous acid conditioning. Cells were grown in complete culture medium for 24, 48 and 72 h. In positive control samples, cells were plated on tissue culture plastic (no dentin slices) and stimulated with 50 ng/mL of bFGF and genomic DNA collected at the same periods. The different symbols indicate mean and the vertical lines indicate the standard deviation of genomic DNA quantity from three independent experiments, measured in triplicate at each time point (* indicates p<0.05 control *vs* 50 ng/mL bFGF, # indicates p<0.05 control *vs* 10 ng/mL bFGF, and ! indicates p<0.05 10 ng/mL bFGF vs 50 ng/mL bFGF)

Next, we assessed the effects of bFGF on the proliferation of the different cell types. This assessment was performed indirectly, by quantifying genomic DNA. In these experiments, we also determined the effect of the culture substrate by comparing the quantity of genomic DNA of cells cultured on cell culture-treated plastic or onto dentin slices. bFGF treatment of periodontal ligament fibroblasts grown on plastic substrate significantly increased cell proliferation at 72 h. When these fibroblasts were grown onto dentin slices, topical application of bFGF also increased cell proliferation in comparison to non-treated dentin substrate, but interestingly, there was no dose-dependent effect. Proliferation of PDL fibroblasts on acid conditioned-dentin was increased at 24 and 48 h and then reduced at 72 h in comparison with nonconditioned dentin (Figure 2A).

Proliferation of cementoblasts on plastic substrate was increased by bFGF at 48 h and subsequently reduced in comparison to vehicle control at 72 h. Similarly to the PDL fibroblasts, proliferation of cementoblasts grown on dentin slices was enhanced by topical application of bFGF, with the lower concentration producing greater increases in cell proliferation (Figure 2B). Proliferation of BMSC cells grown on plastic substrate was significantly increased by bFGF at 48 and 72 h. However, when plated onto dentin substrates, only the higher concentration of bFGF increased proliferation of BMSC on acid-conditioned dentin, whereas in non-conditioned dentin topical application of bFGF inhibited proliferation of BMSCs (Figure 2C).

### Effect of bFGF and culture substrate on gene expression of Runx2, Col1a1, Bglap and Fn

Since bFGF has a range of biological effects beyond cell proliferation, we then assessed its effects on the expression of selected target genes related with the repair/regeneration of periodontal tissues, taking into consideration the nature of the culture substrate. All the gene expression experiments were carried out using 50 ng/mL of bFGF. Cells plated directly onto plastic culture-treated plates served as a positive control to determine the biological effect of this growth factor on target gene expression in each cell type. To account for the effect of the dentin substrate, the negative control was represented by the data on normalized gene expression of cells plated onto nonconditioned dentin slices that were not treated with the growth factor (0 ng/mL of bFGF, control). Results of the experiments in which cells were plated onto dentin slices treated with bFGF are presented according to the cell type and analyzed in terms of fold change of normalized target gene expression in comparison with the expression in the negative control cells. Target gene expression for positive control samples was calculated as a fold change of the expression in untreated cells (0 ng/mL of bFGF) also plated onto tissue culture-treated plastic.

### Cementoblasts (OCCM cells)

**Figure 3:**
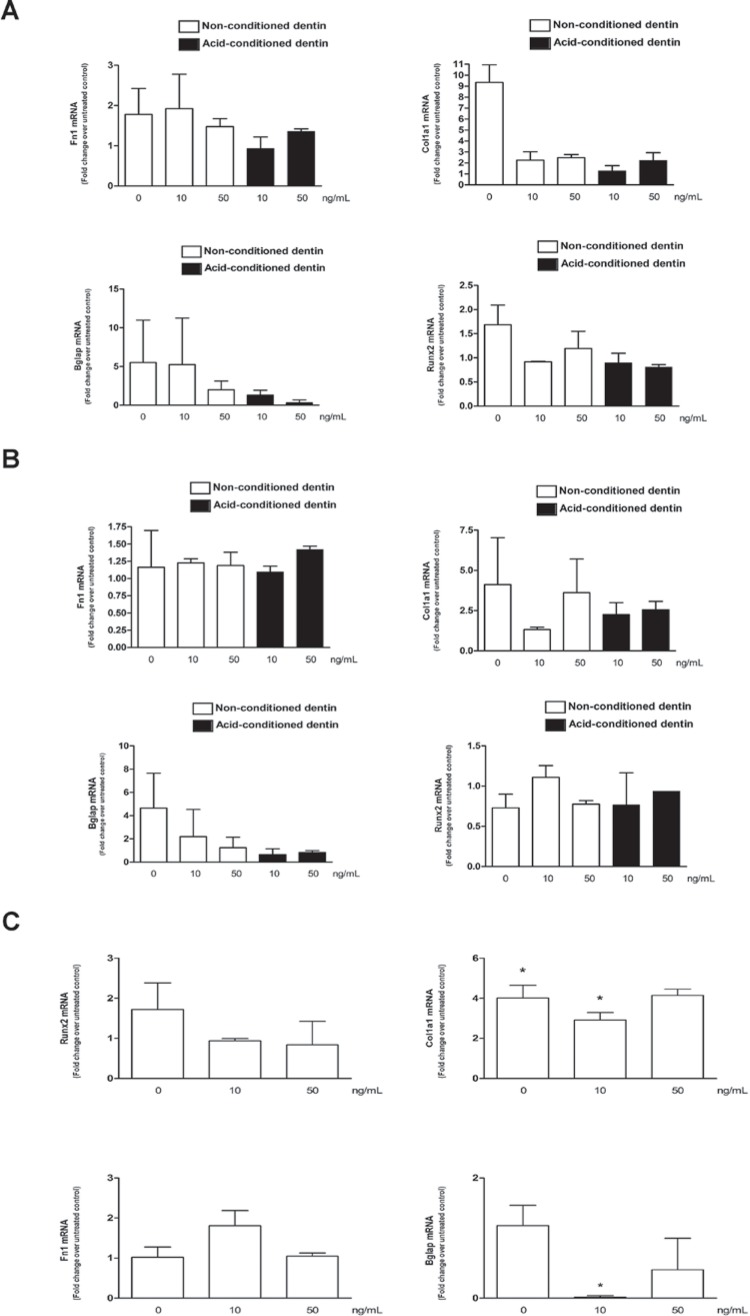
Relative changes in gene expression (mRNA) of Runx2, Col1a1, Fn and Bglap by cementoblasts (OCCM, panel “A”), periodontal ligament fibroblasts (mPDL, panel “B”) and bone marrow stromal cells (BMSC, panel “C”) cultured on different substrates: tissue culture-treated plastic, non-conditioned (NC) and conditioned (C) dentin slices. The same quantity of cells (5×104) was seeded onto the different substrates and total RNA collected after 24 hours. Gene expression of the indicated target genes was determined by RT-qPCR. Bars indicate the mean result of the relative change (fold regulation) of normalized target gene expression in comparison with vehicle-treated samples in each substrate. Bars indicate mean and vertical lines indicate standard deviation of a minimum of three independent experiments

bFGF increased expression of all target genes, but more markedly of Bglap and Col1a1. When these cells were plated onto dentin substrate, the only significant difference observed was a higher expression of Col1a1 in cells plated onto non-conditioned dentin treated with 50 ng/mL of bFGF in comparison with cells plated onto conditioned dentin and treated with the same concentration of bFGF. Overall, the acid conditioning and concentration of bFGF had no marked effect on target gene expression in these cells (Figure 3A).

### Periodontal ligament fibroblasts (PDL cells)

Expression of all genes studied, except Bglap, was higher when cells were grown onto acid-conditioned dentin slices in comparison with cells grown on nonconditioned dentin. Moreover, there was no dose-dependent effect of bFGF on target gene expression, independently of the acid conditioning of dentin substrate. bFGF treatment of cells plated onto tissue culture-treated plastic increased expression of Col1a1 and Bglap, but not of Fn and Runx2 (Figure 3B).

### Bone Marrow Stromal Cells (BMSCs)

Stimulation of these cells with bFGF significantly increased the expression of collagen, but not of the other target genes. We did not detect target gene expression in BMSCs cultured onto acid-conditioned dentin slices, in spite of comparable cycle thresholds (Cts) for the expression of the housekeeping gene (Gapdh). In non-conditioned dentin slices, the lower concentration of bFGF (10 ng/mL) significantly inhibited expression of Bglap by BMSCs; but significantly increased expression of Fn and Col1a1, whereas the higher concentration of bFGF (50 ng/mL) only increased the expression of Col1a1. There were opposite effects of the dose of bFGF on the expression of Col1a1 (higher for 50 ng/mL of bFGF) and Fn (higher for 10 ng/mL of bFGF) (Figure 3C).

## Discussion

In this study we evaluated, *in vitro*, topical application of bFGF on dentin surface with and without previous acid conditioning, assessing the release of bFGF from dentin and its effects on cell proliferation and on the expression of selected genes associated with the formation/regeneration of soft and mineralized periodontal tissues. These assessments were carried out in three different cell types that may have access to the root surface in the clinical situation and which are also shown to be important for the repair/regeneration of periodontal tissues: periodontal ligament fibroblasts, cementoblasts and bone marrow stromal cells.

We used bovine dentin as a substrate to simulate the clinical situation of treating an osseous defect associated with periodontal disease progression. Bovine teeth were used because they may be easily obtained in large quantities, with good condition and have a more uniform composition^26^ ^,^ ^27^. Yassen, et al.^27^ (2011), discussed the issue of inconsistent data reported with the use of bovine teeth in studies focusing on caries and dental erosion/abrasion; however the same authors comment on the comparison between micro-morphological structure, chemical composition and physical properties of human and bovine dentin; which indicated very similar characteristics. It is important to consider the influence of the substrate on the biological effects and bioavailability of topically applied growth factors, since the substrate may bind to the growth factor reversibly or irreversibly and it may also alter the chemical structure of the topically applied polypeptide, affecting its biological activity. In the clinical situation associated with surgical periodontal treatment, the tooth surface is very likely to be completely devoid of cementum due to the mechanical treatment performed to remove soft and mineralized bacterial deposits.

Acid conditioning of the root surface previously to topical application of bFGF significantly increased the release of bFGF into aqueous-based culture medium. This may suggest that chemical conditioning may prolong the release of topically applied growth factors to the surrounding microenvironment, which could favor their bioactivity considering the short half-life of most growth factors^10^ ^,^ ^22^. On the other hand, it is also possible that the bFGF adsorbed and not released into the culture medium remains on the dentin substrate where it may still exert its biological functions. Comparison between concentration of bFGF added directly to the culture medium in control samples with the concentration of bFGF present in the culture medium of the test samples (in which the growth factor was applied topically onto the dentin slices) indicates that most of the growth factor applied topically is either retained on the dentin substrate or lost/degraded, regardless of chemical conditioning previous to the topical application. The rationale for chemical conditioning includes exposure of the organic matrix, which may favor the adsorption of the topically applied growth factors. This is supported by the fact that after 8 h the concentration of bFGF in the culture medium incubated with preconditioned dentin samples was greater than that in control samples. Also, it is important to consider the possibility of dentin matrix substrate-derived factors released by the chemical conditioning on the cells in the microenvironment; which is supported by the report demonstrating increased mineralization and expression of mineralization-associated genes (bone sialoprotein and alkaline phosphatase, for example) by odontoblasts stimulated with proteins in demineralized dentin extracts^13^.

Clinically, immediately after completion of treatment the exposed root surface is covered by a blood clot and cells derived primarily from the adjacent bone marrow, particularly when some type of mechanical barrier has been interposed between the gingival tissues and the bone as in the guided tissue regeneration technique. However, other cell types in the microenvironment, including periodontal ligament fibroblasts and cementoblasts may also gain access to the area, and there is evidence of an important role for these cell types in periodontal regeneration^5^ ^,^ ^20^.

In fact, probably the main reason for the low predictability and high variability of results in periodontal regeneration is the fact that the attachment unit is composed of various tissues, which are produced by different cell types. Thus, it is not sufficient to obtain bone filling of the defect if this bone is not attached to cementum on the surface of the root via a functional periodontal ligament. We have previously reported that topical application of bFGF influences the morphology and density of fibroblasts grown on dentin, and particularly that dentin conditioning has a positive effect on these parameters^22^. Our present results indicate that chemical conditioning of dentin substrate increased PDL fibroblast cell proliferation in comparison with non-conditioned dentin only at 24 and 48 h, with a reversal in this effect at 72 h. We also observed a significant increase in genomic DNA content of periodontal ligament fibroblasts, representative of increased cell number, 72 h after bFGF stimulation, with no changes detected at 24 or 48 h. In a previous study using primary human periodontal ligament fibroblasts^23^ we also did not observe changes in cell proliferation after a 24 h-stimulation with 1 and 10 ng/mL of bFGF. In this report we expand these findings in fibroblasts to show that bFGF also enhances proliferation of cementoblasts at 48 h and bone marrow stromal cells at 48 and 72 h.

Increased proliferation of these relevant cell types is important to support the repopulation of the substrate/wound area and favor the repair/regeneration; however it is also critical to consider other biological effects of bFGF, particularly on cell differentiation and matrix production. In a previous study^23^, we report that 10 ng/mL of bFGF inhibited expression of Col1a1 by human periodontal ligament fibroblasts, whereas in the present study using a murine periodontal ligament fibroblast cell line and a higher concentration of bFGF (50 ng/mL) we only observed a discrete increase in Col1a1 expression. Interestingly, 50 ng/mL of bFGF strongly induced Col1a1 gene expression by cementoblasts and also in BMSC cells, albeit less potently.

When bFGF was applied on the dentin substrate, Col1a1 expression was increased in PDL fibroblasts grown on acid-conditioned dentin, but not in nonconditioned dentin; whereas in BMSC cells the increase in Col1a1 gene expression was significant and dose-dependent, but only in non-conditioned dentin. However, cementoblasts were less responsive to bFGF in terms of increase in Col1a1 expression, particularly when grown on acid-conditioned dentin.

bFGF stimulation of all three cell types had little effect on the expression of Fn and Runx2. The most interesting findings were the greater increase in Fn expression by BMSC cells grown on non-conditioned dentin exposed to the lower concentration of bFGF and the fact that PDL fibroblasts presented a marked increase in the expression of both Fn (without dose-dependent effect) and Runx2 (inverse dose-dependent effect) only when grown in acid-conditioned dentin.

Cementoblasts were the least responsive cell type, as the expression of Fn, Runx2 and Bglap by OCCM cells were not markedly regulated by bFGF in any of the experimental conditions. Notably, BMSCs grown on conditioned dentin had barely detectable levels of mRNA for all target genes (data not shown). This suggests that chemical conditioning may render the substrate unfavorable for cell metabolism of BMSCs. In fact, we cannot rule out a cytotoxic effect in acid-conditioned dentin, as the proliferation was assessed by genomic DNA quantitation without verification of DNA integrity or of cell viability by other methods.

It is important to note that in the positive control samples (cells grown directly on tissue culture plastic, without dentin slices) the concentration of bFGF was much greater than in the experimental samples. This was due to the fact that we used a defined volume of bFGF for the topical application, whereas in the positive control samples (cells grown on tissue culture plastic) we used a defined concentration (ng/mL) of the same growth factor. We note that it is not possible to “topically apply” or “condition” tissue culture plastic with the growth factor, similarly to what was done with the dentin slices. Also, even though we seeded the cells using the same protocol in both cell culture plastic and dentin slices to assure the exact same n = initial numbers of cells and also a similar cell density, it is possible that the cells grown in tissue culture plastic proliferated faster than those plated onto dentin slices, resulting in different cell numbers at the end of the experimental period. We speculate that this possible difference in cell proliferation is influenced primarily by the differences in substrate composition rather than by the initial cell numbers and cell density. Anyway, these considerations preclude direct comparison between the results from positive control and experimental groups (cells grown on dentin slices), but the information provided by the negative control samples (treated with the vehicle used to resuspend the bFGF) indicates that bFGF used was biologically active and the information provided by the positive control samples (cells grown on tissue culture plastic and treated with bFGF) informs the general trend for the effects on the expression of the target genes in the different cell types. Nevertheless, we have not determined how much of the initial quantity of the topically applied bFGF was retained/adsorbed on the dentin substrate, nor have we directly confirmed that the growth factor eventually present on the dentin surface retained its full biological activity, but we consider the modulation of gene expression and cell proliferation as indirect evidence of bFGF bioactivity.

In summary, we show that previous acid conditioning of dentin improves the release of topically-applied bFGF in aqueous medium. We have also shown that different cell types that are relevant for periodontal regeneration respond differently to bFGF; however there was an overall increase in cell proliferation and in the expression of bone and connective tissue matrix gene expression by cells grown on dentin with bFGF. Cementoblasts were the most responsive cell type in terms of regulation of gene expression by bFGF in the absence of a dentin substrate; whereas PDL cells were most responsive to bFGF in terms of gene expression when grown on acid-conditioned dentin. Although no clear-cut conclusion may be drawn from our results, the data indicate that topical application of bFGF has biological effects on relevant cell types for periodontal regeneration and warrants *in vivo* studies to fully assess the potential of this approach on periodontal regeneration.
